# Evidence of friction reduction in laterally graded materials

**DOI:** 10.3762/bjnano.9.229

**Published:** 2018-09-13

**Authors:** Roberto Guarino, Gianluca Costagliola, Federico Bosia, Nicola Maria Pugno

**Affiliations:** 1 Laboratory of Bio-Inspired & Graphene Nanomechanics, Department of Civil, Environmental and Mechanical Engineering, University of Trento, Via Mesiano 77, 38123 Trento, Italy; 2 Department of Physics and Nanostructured Interfaces and Surfaces Centre, University of Torino, Via Pietro Giuria 1, 10125 Torino, Italy; 3 Ket Lab, Edoardo Amaldi Foundation, Italian Space Agency, Via del Politecnico snc, 00133 Rome, Italy; 4 School of Engineering and Materials Science, Queen Mary University of London, Mile End Road, E1-4NS London, United Kingdom

**Keywords:** bio-inspired materials, friction, functionally graded materials, numerical simulations

## Abstract

In many biological structures, optimized mechanical properties are obtained through complex structural organization involving multiple constituents, functional grading and hierarchical organization. In the case of biological surfaces, the possibility to modify the frictional and adhesive behaviour can also be achieved by exploiting a grading of the material properties. In this paper, we investigate this possibility by considering the frictional sliding of elastic surfaces in the presence of a spatial variation of the Young’s modulus and the local friction coefficients. Using finite-element simulations and a two-dimensional spring-block model, we investigate how graded material properties affect the macroscopic frictional behaviour, in particular, static friction values and the transition from static to dynamic friction. The results suggest that the graded material properties can be exploited to reduce static friction with respect to the corresponding non-graded material and to tune it to desired values, opening possibilities for the design of bio-inspired surfaces with tailor-made tribological properties.

## Introduction

Materials with a gradient in their physical or elastic properties are widely found in nature. Several known biological systems have developed specialized functionalities due to stiffness, density or composition gradients. Beetles, for instance, display setae with a graded stiffness that optimises the adhesive performance on rough surfaces [1]. Hardness and stiffness gradients are of fundamental importance in the biomechanics of contacts, since they allow increased resistance against wear, impact, penetration and crack propagation [2–7]. Bio-inspired solutions have thus been proposed for the design of advanced materials that mimic the hierarchical and graded structures found in nature, for use in engineering applications [8–9].

Functionally graded materials (FGMs) display a gradient in their elastic properties along one or more directions and have recently acquired great interest in technology [10]. Several authors have studied standard solid mechanics problems considering FGMs, for example, in the case of various loading conditions [11–13], and in problems involving fracture [14–17] or fatigue [18].

Recently, FGMs have also been applied to tribological studies, where it is well known that the behaviour of a system is governed by multiphysics and multiscale interactions [19]. The first application of graded materials to contact mechanics was proposed by Giannakopoulos and Suresh, who presented an analytical study of the indentation of materials with an exponential or power law variation of the Young’s modulus through the depth [20–21]. Giannakopoulos and Pallot then extended the analysis to 2D [22]. Graded substrates have also been considered in elastohydrodynamic lubrication problems [23]. More recently, the method of dimensionality reduction [24–25] has been extended to the axisymmetric frictionless contact of elastically graded materials [26], and solutions are also provided in the presence of adhesion [27]. In all these cases, the elastic gradients are considered with respect to the depth, with an exponential or a power law variation of the Young’s modulus, i.e., *E*(*z*) = *E*_1_e^α^*^z^* or *E*(*z*) = *E*_2_*z*^β^, respectively, where *z* is the depth coordinate and *E*_1_, *E*_2_, α and β are constants. The first extension to a lateral elastic gradient, to the best of our knowledge, was by Dag et al*.* who studied the problem both analytically, by reducing the equation describing the contact of a rigid flat punch to a singular integral equation, and numerically, through the finite-element method [28–29].

In this paper, we extend the previous work on 1D composite surfaces [30] to 2D geometries to show how it is possible to tune the macroscopic tribological properties through local variations of material and surface properties, i.e., Young’s moduli and friction coefficients, reducing static friction compared to the non-graded case. The results also allow the predictions of a discrete approach like the spring-block model [31–32] to be compared to those derived by explicit finite-element simulations. This provides useful insights to understand the frictional properties of graded materials, with the aim of designing smart tribo-materials and innovative solutions for sliding interfaces.

## Methods

### Introduction

In this work, we investigate the effect of surface or material property gradients on the global coefficient of friction. The system taken into consideration is composed of an elastic plate, with a square base of side *L* and height *H* << *L*, which is driven from the top surface at constant velocity over a rigid substrate and subjected to friction. We study this system by means of two numerical methods: a 2D spring-block model (SBM) and 3D finite-element method (FEM) simulations. The two methods are complementary in many aspects, so that by using both it is possible to cross-check the results and obtain interesting insights from different approaches.

The SBM is a two-dimensional approximation of the real system, so that effects due to the thickness of the layer are neglected. Specifically, any effect due to the vertical stress distribution cannot be captured. While these can be minimized in the case *H* << *L*, it is still useful to compare the results with FEM simulations, which can model this thin layer while maintaining a 3D approach. As we will show later, the comparison between the two methods will allow some concurrent effects to be disentangled that govern the global frictional behaviour. On the other hand, different formulations of SBM have been used in many recent studies to describe aspects of the transition from static to dynamic friction, the nucleation of rupture wave fronts, and the effects of patterning [32–36]. The SBM method is usually computationally faster than FEM, thus it is more practical for a qualitative understanding of these phenomena, but also includes approximations that must be verified to check whether all effects are correctly described. Thus, in each section, we will consider the two models with the same setup, that is, by choosing the closest conditions and parameter sets for the two approaches, and we will describe the effects predicted by them in the presence of graded materials.

### 2D spring-block model

**Figure 1:**
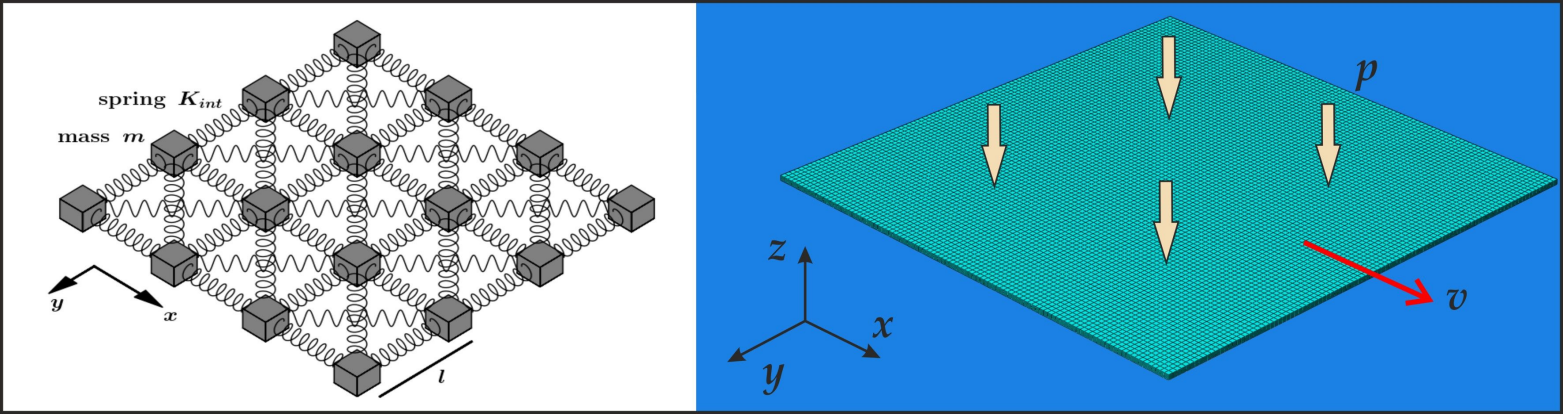
Schematic of the numerical models used in this work. Left: 2D discretization in springs and masses used in the SBM approach. Right: 3D discretization of a deformable plate (green) sliding on a rigid surface (blue), with applied normal pressure and velocity, used in the FEM approach.

We adopt the formulation of the 2D spring-block model introduced in Costagliola et al*.* [32]. The sliding surface is discretised with *N*_b_ = 120 blocks in both the *x* and *y* directions, placed at a distance *l = L/N*_b_. The thickness of the layer is set to *l_z_*, so that the block mass is *m =* ρ*l_z_l^2^*, where ρ is the density of the material. The spring mesh is arranged as shown in Figure 1. In order to obtain the equivalent of a homogeneous elastic material with Young’s modulus *E* and Poisson’s ratio 1/3, the stiffness of the springs along the axis is set to *K*_int_* =* 3/4 *El_z_*, and of the diagonal springs to *K*_int_/2 [37]. Thus, the internal elastic force exerted on the generic block *i* by its neighbour *j* is ***F***_int_^(^*^ij^*^)^* = k_ij_* (*r_ij_* − *l_ji_*) (***r****_j_* − ***r****_i_*) /*r_ij_*, where ***r****_i_* and ***r****_j_* are the position vectors of blocks *i* and *j*, respectively, *r_ij_* is the modulus of their distance, *l_ij_* is their rest distance and *k_ij_* is the stiffness of the spring linking them.

All the blocks are connected to a slider, moving at constant velocity **ν**, through a spring with stiffness *K*_s_. The force exerted on the block *i* by the slider is ***F***_s_^(^*^i^*^)^* = K*_s_ (**ν***t* + ***r****_i_*^0^* −* ***r****_i_* ), where ***r****_i_*^0^ is the initial position of the block and **ν** is the velocity vector of the slider, e.g., **ν*****=* (ν,0) when sliding is along the *x* axis. Therefore, the total driving force acting on the block *i* is ***F***_mot_^(^*^i^*^)^ *= ****F***_s_^(^*^i^*^)^* + Σ_j _****F***_int_^(^*^ij^*^)^.

A damping force 
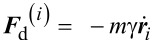
 is added to avoid artificial block oscillations, where γ is the damping coefficient and 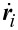 is the velocity vector of the block. The damping coefficient γ is an arbitrary parameter. The results are independent of its value provided it is fixed in the underdamped regime: 
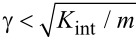
 [34]. A pressure *p* is applied on the whole system, so that on each block there is normal force *F*_n_^(^*^i^*^)^ *= pl^2^*. Hence, the total normal force is *F*_n_* = pL^2^*.

The interaction between blocks and substrate is modelled through the classic Amontons–Coulomb friction force: each block has a static µ_s_^(^*^i^*^)^ and dynamic µ_k_^(^*^i^*^)^ friction coefficient, randomly assigned at the beginning of the simulation from a Gaussian statistical distribution (to account for surface roughness) with mean values denoted with µ_s_^(m)^ and µ_k_^(m)^, respectively. The standard deviation on the local coefficients of friction are denoted with σ_µs_ and σ_µk_, respectively.

If the block *i* is at rest, the static friction force ***F***_fr_^(^*^i^*^)^ opposes the total driving force, so that ***F***_fr_^(^*^i^*^)^ = −***F***_mot_^(^*^i^*^)^ , up to the threshold value *F*_fr_^(^*^i^*^)^ = µ_s_^(^*^i^*^)^ *F*_n_^(^*^i^*^)^. When this threshold is exceeded, a constant dynamic friction force with modulus *F*_fr_^(^*^i^*^)^ = µ_k_^(^*^i^*^)^* F*_n_^(^*^i^*^)^ opposes the motion.

Thus, Newton’s equation of motion for the block *i* can be written as 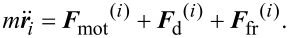 The overall system of equations is solved with a fourth-order Runge–Kutta algorithm. The simulation is repeated many times, extracting each time new friction coefficients from the statistical distributions, for statistical reliability. An integration time step of 10^−8^ s is sufficient to reduce the time integration error under the statistical variability. Various observables can be calculated from the solution, for example, the total tangential force, which is the modulus of the sum of the forces exerted by the slider and corresponds to the macroscopic friction force ***F***_fr_ = |∑*_i_****F***_s_^(^*^i^*^)^*|.*

For further information and the discussions of the influence of the parameters, we refer the reader to our previous work [32].

### 3D finite-element model

3D explicit FEM simulations are carried out for a deformable plate sliding on a rigid flat surface. Each simulation is performed in two steps: first, a constant pressure is applied to the top surface of the block, increasing linearly from zero to the nominal value, in order to create the nominal area of contact; then, a constant velocity is applied instantaneously to the same top surface of the block. The rigid surface is fixed with a 3D clamp in order to constrain all its degrees of freedom. The complete setup is schematised in Figure 1.

The sliding block is discretised with 100 elements both in *x* and in *y* directions, and with 5 elements along the thickness, for a total of 50,000 hexahedral elements. The simulations are performed using Abaqus^®^ (version 6.13, Dassault Systèmes, France) and employing C3D8I elements, which are 8-node bricks with 8 points of integration and incompatible modes. The choice of this element type allows a good representation of the stress singularities at the edges (see Supporting Information File 1). A convergence study is carried out by monitoring the total strain energy of the system, to choose a sufficiently fine discretization.

We assign a velocity-dependent coefficient of friction to the contact surfaces, evolving as:

[1]
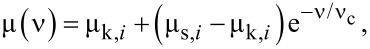


where µ_s,_*_i_* and µ_k,_*_i_* are the static and dynamic local friction coefficients, respectively, ν is the sliding velocity, and ν_c_ is the critical velocity for the transition [38]. This expression ensures that the value of the dynamic coefficient of friction is reached only when the sliding velocity is sufficiently high (i.e., ν >> ν_c_). The static friction threshold is retrieved for ν = 0, so that the friction force approximates the classic Amontons–Coulomb friction force adopted in the SBM model. The contact between the block and the rigid surface is implemented through a surface-to-surface formulation using the penalty contact method [39]. Here, as opposed to the SBM, the local coefficient of friction is constant over the corresponding contact area and no statistical dispersion is introduced. The global coefficients of friction are finally calculated by dividing the resulting total lateral force by the applied normal force.

### System parameters

We consider an elastic plate sliding on a rigid flat surface. The block has a square area of side *L* = 5 mm and thickness *H* = 0.05 mm. We consider a linear elastic material, with density ρ = 1.2 g/cm^3^, Poisson’s ratio 1/3 and a reference Young’s modulus *E* = 10 MPa (i.e., the reference value around which the gradients are implemented). We adopt typical values for the applied pressure of *p* = 10 kPa and for the sliding velocity ν = 1 mm/s, keeping their value constant for all the simulations.

The reference values of the local static and dynamic coefficients of friction are µ_s,_*_i_* = 1.0 and µ_k,_*_i_* = 0.6, respectively. In the SBM model, these are the mean values of the Gaussian distribution, i.e., µ_s_^(m)^ and µ_k_^(m)^, respectively.

**Figure 2:**
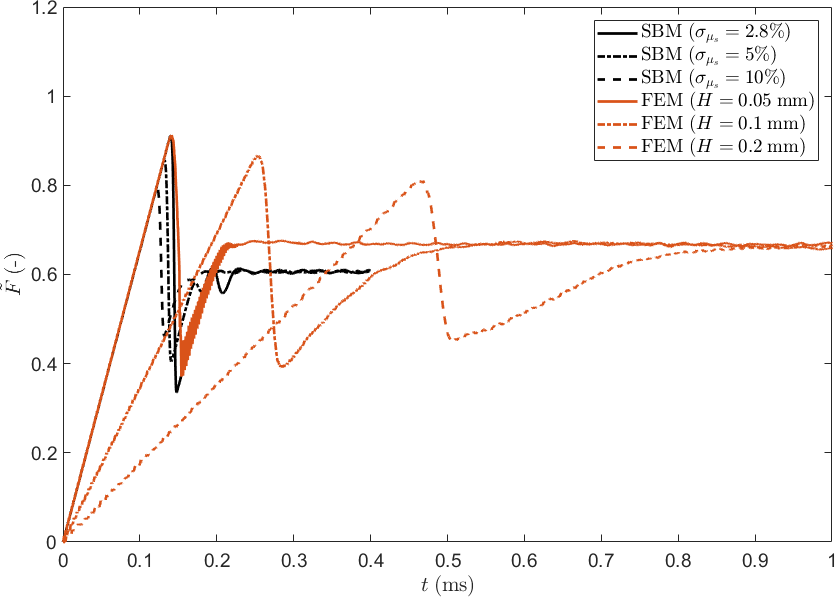
Dimensionless friction force 
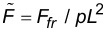
 as function of time for the non-graded material: SBM solution for different values of dispersion σ_μs_ of the local static coefficient of friction (black lines) and FEM solution for different block thicknesses *H* (red lines).

In Figure 2, we show the typical time evolution of the tangential force obtained with the SBM for various σ_µs_. The maximum of the friction force decreases when increasing the statistical dispersion, as in the case of the 1D formulation [30]. The time evolution obtained with FEM is also shown. In this case, the behaviour is strongly dependent on the thickness of the block. The time interval Δ*t*_s_ needed to reach the static friction peak can be estimated starting from the shear stress τ = *G*γ, where *G* is the shear modulus. If the shear deformation is γ = νΔ*t*_s_/*H*, the static friction peak is reached when τ*L^2^* = µ_s_*pL^2^*, i.e. for:

[2]
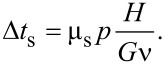


Simulations indicate that with a standard deviation of σ_μs_ = 2.8% a thickness *l* = 0.057 mm and *H* = 0.05 mm, SBM and FEM results coincide in the case of a uniform non-graded surface. This parameter set is the reference case for the following comparisons.

We thus investigate the effects due to a grading of the material properties. Denoting a generic material property with φ, the corresponding linear gradient is described by:

[3]
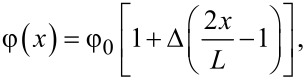


where φ_0_ is the reference value (i.e., relative to the non-graded system) and Δ is the maximum variation at the edge. Discretizing the length into *N* homogeneous parts, Equation 3 can be written as:

[4]
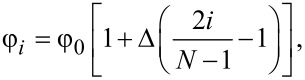


Therefore, the linear gradient is approximated with a stepwise function. For simplicity, we study a linear gradient instead of the power-law variation usually considered in the literature (see, e.g., [20,22]). This does not entail any loss of generality, since, as discussed below, the macroscopic frictional behaviour is determined mainly by the overall variation in the considered property between the edges and the centre of the plate.

In addition, we also consider triangular gradients, which are described by:

[5]
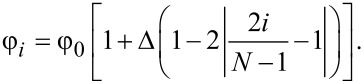


**Figure 3:**
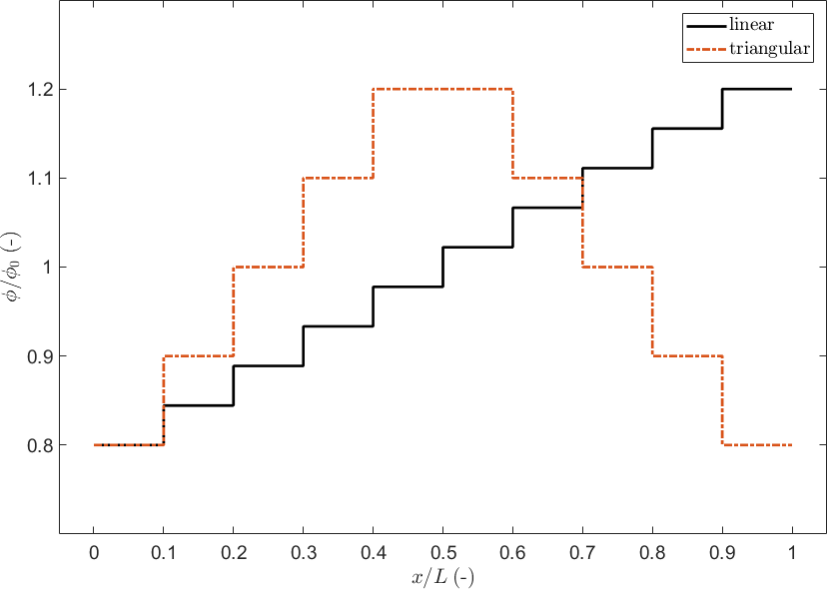
Examples of stepwise linearly increasing and triangular property gradients considered in this work for the case Δ = 0.2.

This gradient is therefore positive when φ is larger at the centre and negative when φ is larger at the borders. Figure 3 shows examples of the considered stepwise linearly increasing and triangular property gradients.

For convenience, the gradients are implemented for *N* = 10, so that the surface is actually divided into bands perpendicular to the *x* axis. Simulations with the SBM indicate that the results were relatively insensitive to *N*, including in the case *N* = *N*_b_, since small variations due to a very fine discretization are concealed by the statistical dispersion of the local friction coefficients. A reduced influence of *N* is also found in the FEM simulations, which are much more sensitive to the variation of the overall gradient (i.e., Δ), than to the discretization step. Thus, in each region of the gradient discretization, the value φ*_i_* is assumed constant and the model parameters are defined according the general definitions given above.

## Results and Discussion

### Gradient in the local coefficients of friction

We first consider a gradient in the local friction coefficients. In real systems, this can be realised in two ways. First, the surface can be polished in a spatially variable manner or using different processes in order to have a varying roughness and thus varying local friction coefficients. Secondly, a surface with a gradient in the frictional properties can be obtained by appropriately fabricating and arranging microscopic structures of variable geometries or sizes, giving rise to variable local friction coefficients [40–41].

In order to compare the results, we report the variations of the global static coefficient of friction as function of a grading distribution Δ in the local coefficient of friction, with respect to the value of the non-graded surface, using both SBM and FEM. The variation is computed as 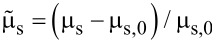, where µ_s,0_ corresponds to the case Δ = 0. The absolute values of µ_s_ are reported in the Supporting Information File 2.

**Figure 4:**
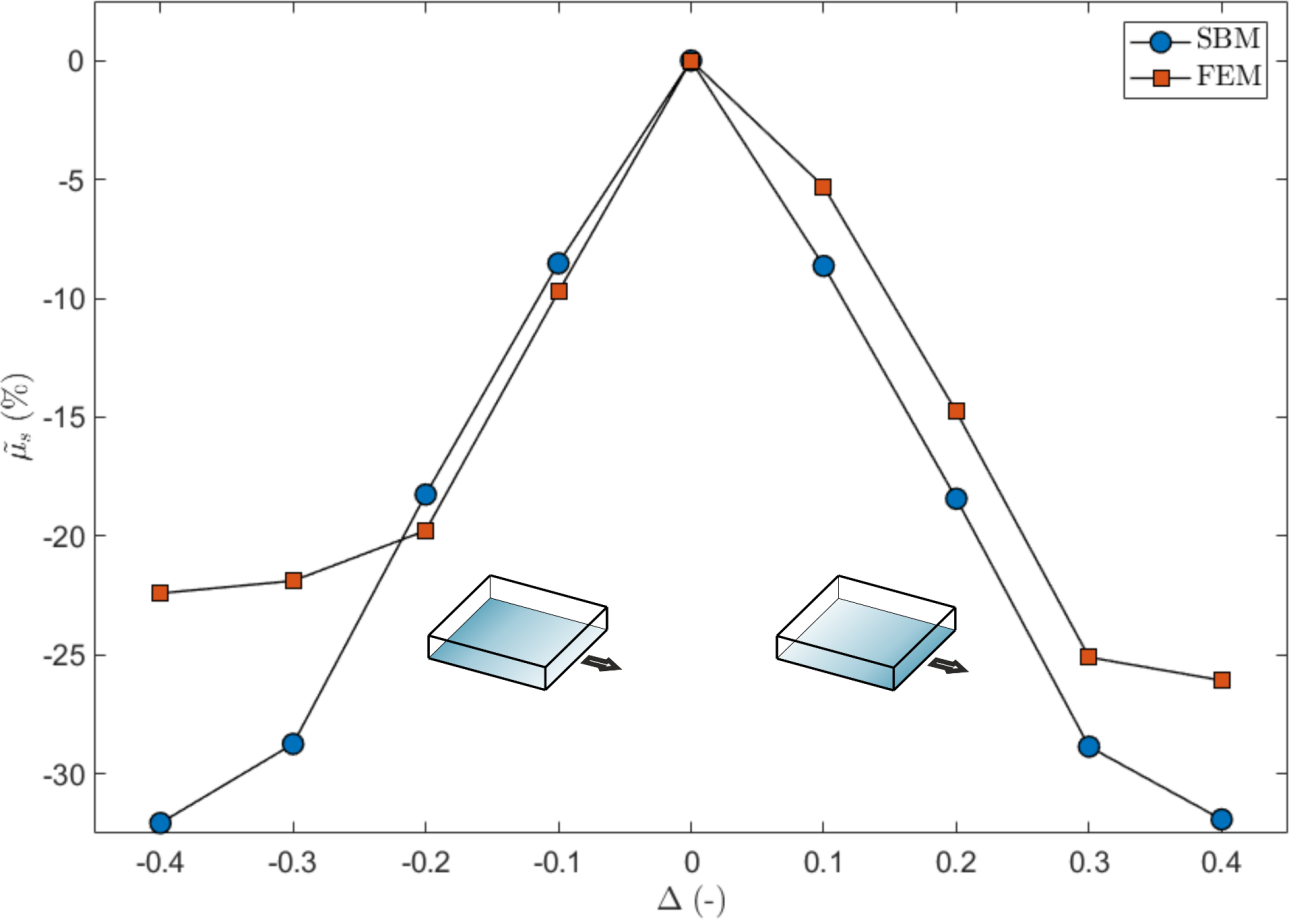
Effect of a gradient in the local coefficients of friction on the global static coefficient of friction, expressed as percentage variation as a function of Δ. The shaded blocks schematically represent the value of the local coefficients of friction, which are higher for a darker shading.

The results are shown in Figure 4 for both SBM and FEM simulations. In general, in the presence of a gradient, the global static coefficient of friction of the surfaces in contact decreases with respect to the non-graded surface, although the mean values of the local friction coefficients are the same.

**Figure 5:**
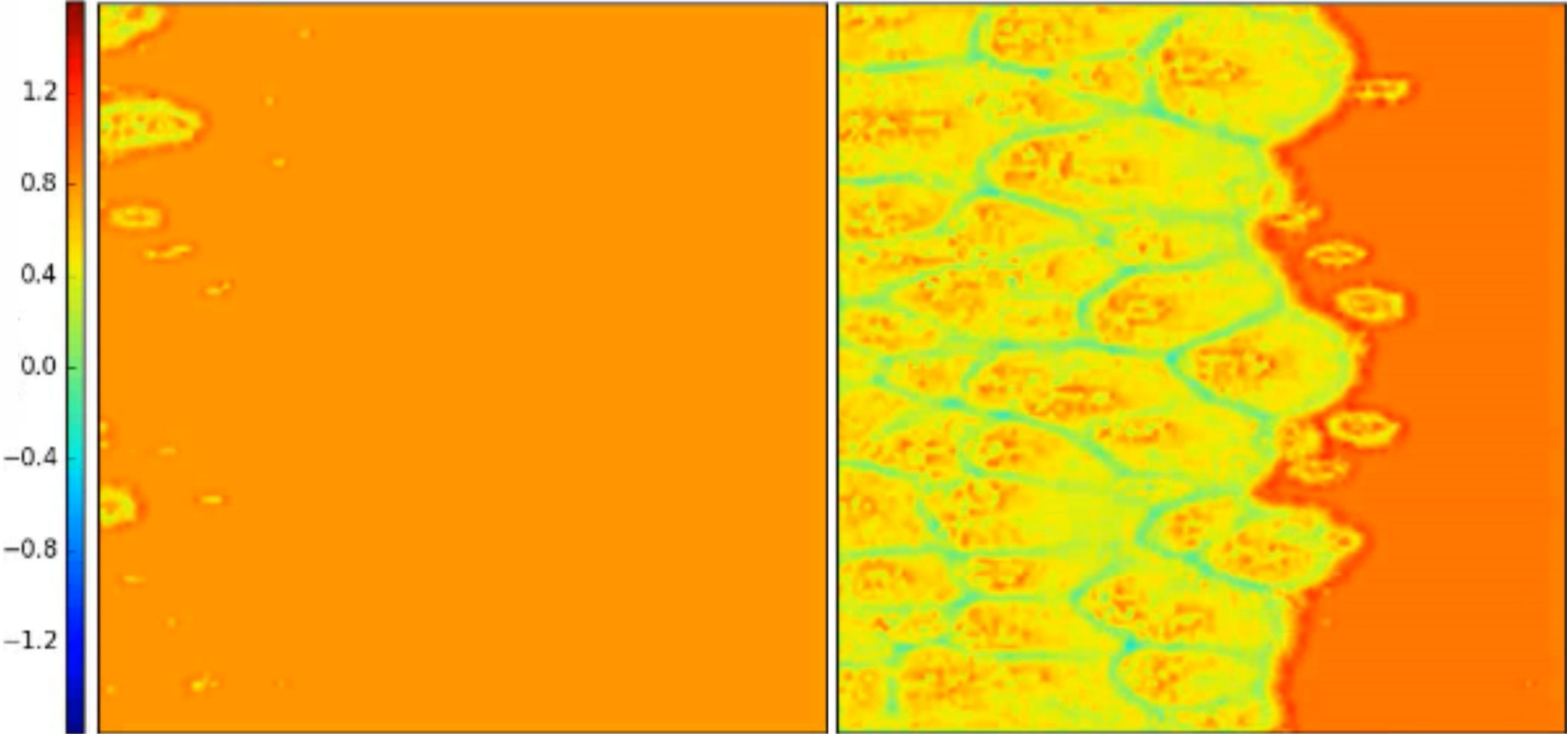
Propagation of the detachment front at the static friction threshold (left) and immediately after (right), in units of the dimensionless longitudinal stress σ*_x_*/*p*, for a surface with a gradient in the local coefficients of friction, computed using the SBM method for the case Δ = 0.1. The irregularities of the detachment front are due to the statistical dispersion of the local coefficients of friction introduced in the SBM formulation.

**Figure 6:**
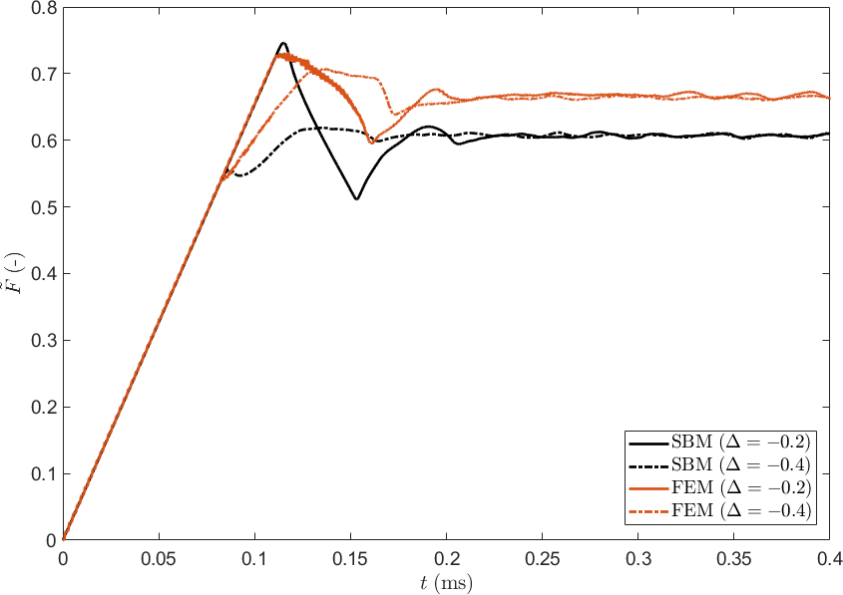
Dimensionless friction force as function of time for two values of Δ, calculated using the SBM and FEM methods. In both cases a larger value of Δ leads to the reduction or elimination of the initial force peak.

The reason for this lies in the progressive detachment of the contact surfaces, always starting from the side where the critical value of the local shear stress is reached (i.e., the static friction threshold). The first detachment of the sliding surface produces a detachment avalanche propagating towards the region with higher static friction threshold, as shown in Figure 5 (see also Supporting Information File 3). Analogous effects on the propagation of detachment fronts have also been studied experimentally [42]. Consequently, an increasing absolute value of Δ reduces the global static coefficient of friction with respect to the non-graded surface, up to an asymptotic value corresponding to the dynamic friction coefficient value. Thus, the gradient can completely remove the force peak observed at the transition from static to dynamic friction (see Figure 2). This is schematically shown in Figure 6, where the time evolution of the friction force is reported for two different values of Δ. An additional effect is the deviation from linearity when approaching the static friction threshold, observed for the highest value of the gradient (i.e., Δ = 0.4) and similarly highlighted by both the SBM and the FEM simulations.

Unlike the SBM simulations, which give symmetric results with respect to the case Δ = 0 since they are insensitive to the vertical stress distribution, FEM simulations display an anisotropic behaviour when considering a positive or a negative gradient. This is equivalent to considering the same sign of the gradient but with an opposite sliding direction. This result can be attributed to the vertical stress distribution at the contact interface: when friction is present, the normal pressure is reduced at the leading edge of the sliding plate and increased at the trailing edge [28,43–44]. Since the static friction thresholds depend not only on the local µ_s_ but also on the local value of the normal pressure, due to this effect, a gradient of detachment threshold already exists. This must be added to the gradient of the local coefficients of friction. As an example, for Δ > 0, the static friction thresholds are increased at the leading edge of the surface, so that the effect of the vertical stress acts as a counterbalance, and the effective gradient is smaller than Δ. Conversely, for Δ < 0, the vertical stress accumulates with the gradient. For this reason, with the same absolute value but different sign of Δ, we can expect a different behaviour; in particular, that for a positive Δ value, the global static friction is greater than for the case of a negative Δ value.

From the results of the FEM simulations, this is reproduced correctly at least for Δ < 0.3, as can be seen in Figure 4. For higher values of the gradient, the results are opposite due to the large difference of friction between the edges. For Δ < 0 at the leading edge of the surface, static friction is already weak so that the effect of normal pressure reduction is less influential, while at the trailing edge, static friction is large due to the combination of a large local friction coefficient and increased pressure. The result is that the detachment process is inhibited with respect to the same positive Δ value.

The effect of larger values of Δ on the detachment process is also investigated through the SBM method. As previously explained, the detachment front nucleates from the edge where the weakest thresholds are, and the maximum of the friction force during the time evolution occurs shortly after the detachment begins. However, when the gradient increases, the time necessary for the detachment front to propagate across the surface increases (see Figure 6 and Supporting Information File 4). For higher values of Δ, the contribution to the total friction force from the region with higher thresholds is more influential, so that the maximum of the friction force occurs later during the detachment process and not shortly after its beginning.

Thus, while SBM cannot capture anisotropic behaviour emerging from 3D deformation effects occurring in the materials in contact, it is still useful to disentangle the effects of the gradient and the vertical stress distribution.

**Figure 7:**
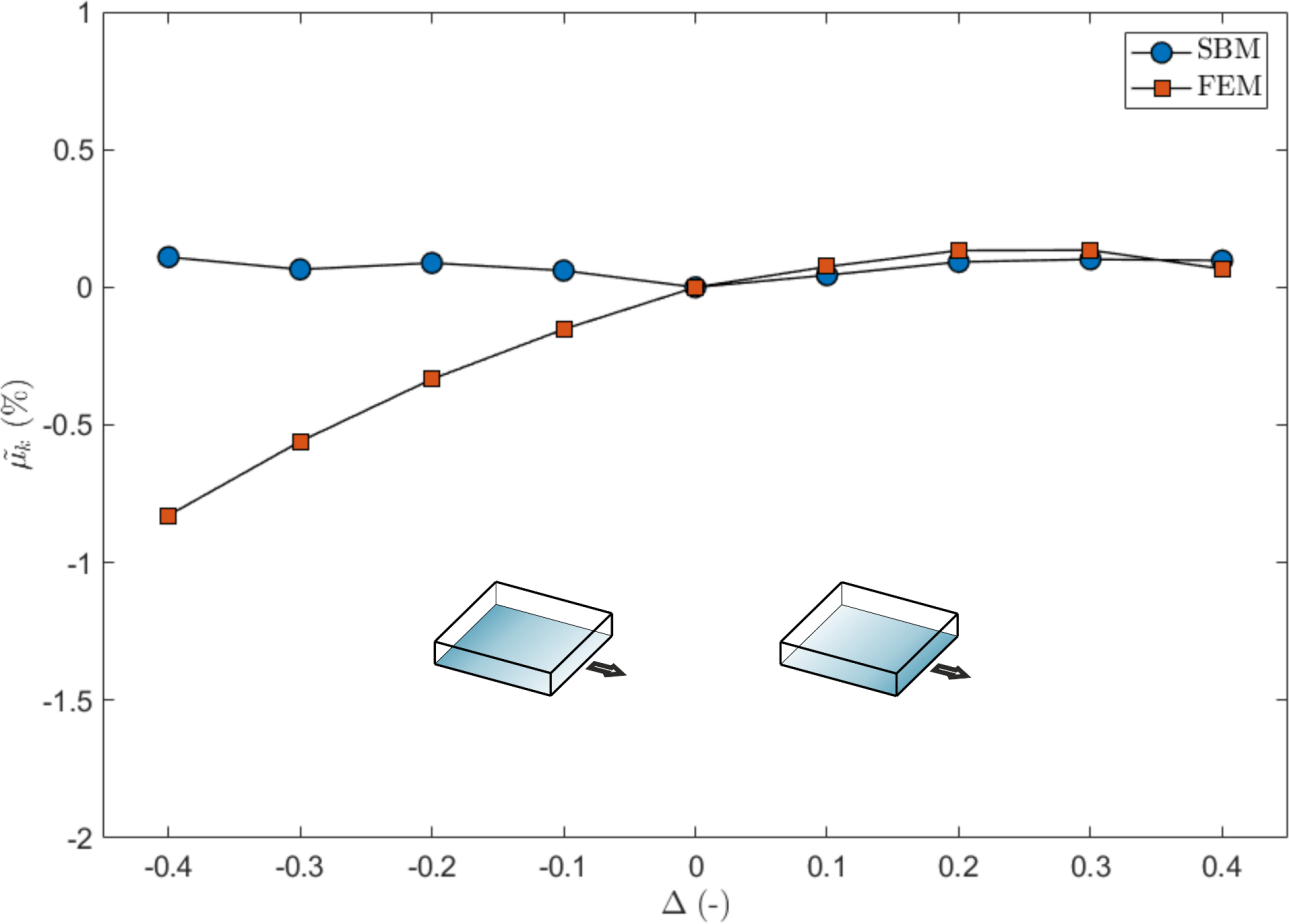
Effect of a gradient in the local coefficients of friction on the global dynamic coefficient of friction, expressed as percentage variation as a function of Δ. The shaded blocks schematically represent the value of the local coefficients of friction, which are greater for darker shading.

The global dynamic friction coefficient µ_k_ does not display any appreciable variation when calculated with FEM or SBM if compared to the flat surface, i.e., its variation is limited to within 1% as shown in Figure 7. Again, the FEM results are anisotropic with respect to Δ, for the reasons explained above. However, the effect of the gradient on the dynamic friction cannot be fully captured by a formulation only based on an Amontons–Coulomb friction law, as in the case of the SBM. Therefore, a good match between the two methods cannot be achieved here and further investigations are needed.

**Figure 8:**
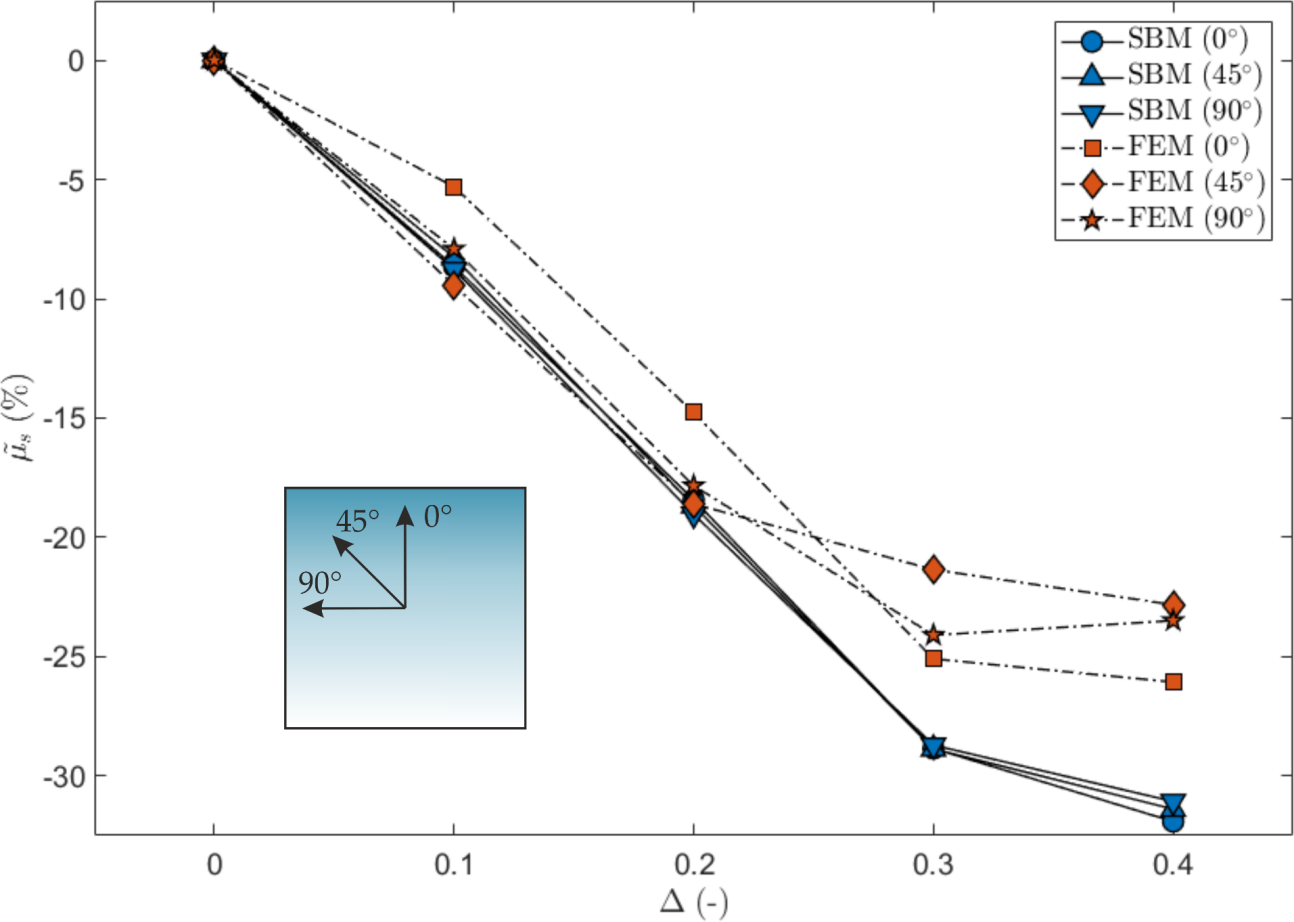
Effect of a gradient in the local coefficients of friction on the global static coefficient of friction, expressed as percentage variation as a function of Δ and for three sliding directions. The shaded square schematically represents the value of the local coefficients of friction, which are greater for a darker shading, and the considered sliding directions.

We have also investigated the effect of changing the sliding direction with respect to the direction of the gradient, as shown in Figure 8. Both the SBM and the FEM predict a greater global static coefficient of friction when switching from the 0° to the 90° direction, and this is evident especially for large values of Δ. The dependence of µ_s_ on the angle, instead, is more complex for Δ < 0.3, especially for the 3D FEM, where the interaction with the vertical stress distribution must also be taken into account, as discussed previously.

### Gradient in the Young’s modulus of the material

**Figure 9:**
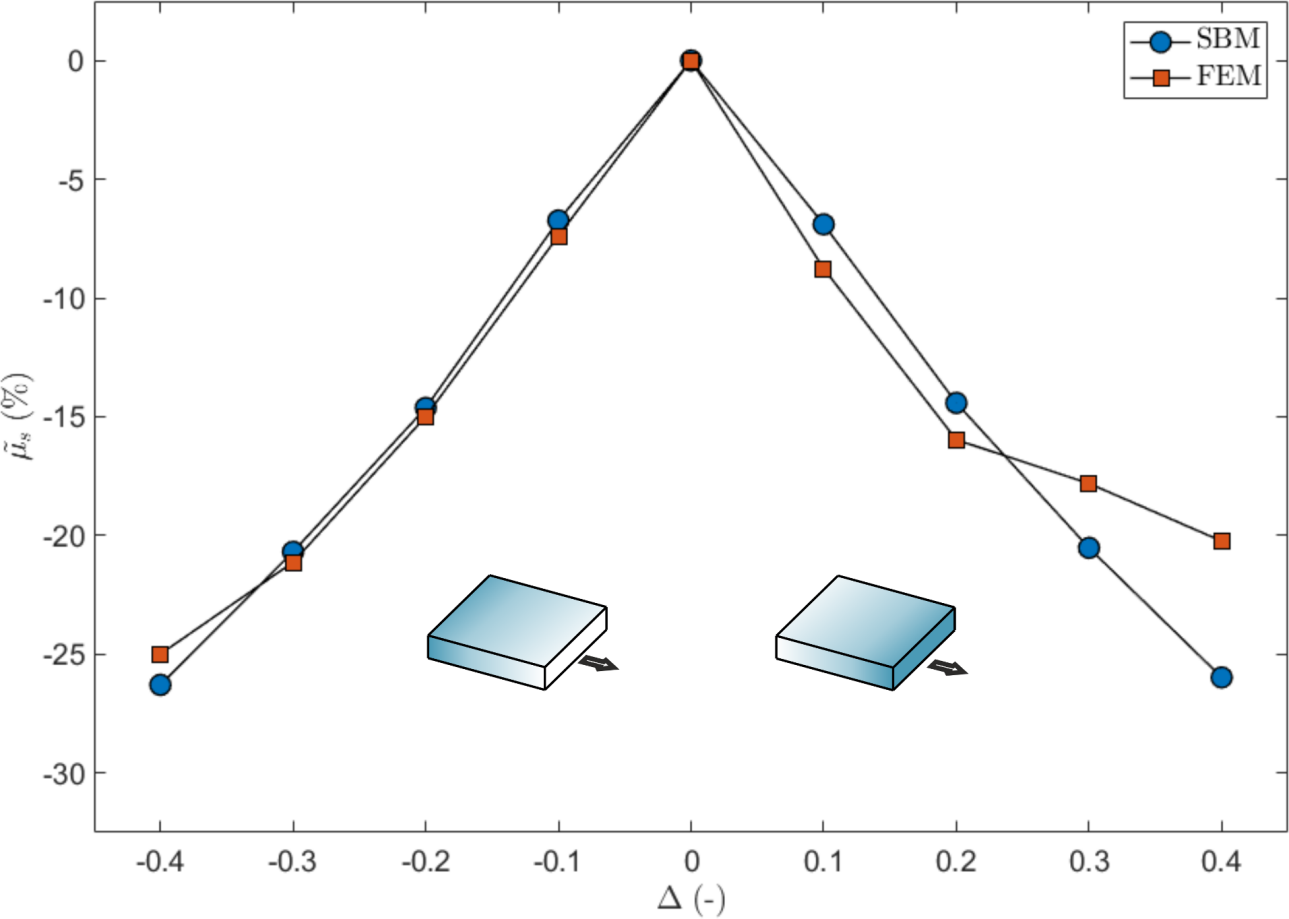
Effect of a gradient in the Young’s modulus on the global static coefficient of friction, expressed as variation as function of Δ. The shaded blocks schematically represent the value of the local Young’s modulus, which is greater for darker shading.

The effect of a gradient in the Young’s modulus is qualitatively similar to that of the graded coefficient of friction discussed above. As can be seen in Figure 9, the global µ_s_ for the graded material is smaller than that for the case Δ = 0. However, while in the previous case, the reason for the modification of the global friction coefficient can be found in a smaller static friction threshold, in this case, a given lateral strain produces a corresponding tangential force that is greater on the side of the material with greater local Young’s modulus. Therefore, in this case, the static friction threshold is reached first where the Young’s modulus is greater. The detachment of the contact surfaces starts from this side and proceeds towards the region with smaller *E*, with a propagation similar to that already shown in Figure 5, but in the opposite direction. Thus, the material portion of the sliding plate is in tension for Δ > 0 and in compression for Δ < 0. Qualitatively speaking, a positive gradient in the Young’s modulus is equivalent to a negative gradient in the local coefficients of friction.

Again, FEM simulations produce an anisotropic result with respect to positive and negative gradients, for the reasons discussed above. The redistribution of normal stresses is again related to the static friction threshold: when Δ > 0, the tangential force is greater at the leading edge of the slider, where *E* is higher and the thresholds are reduced due to smaller *p* values, so that the effective gradient is larger than Δ. Conversely, for Δ < 0 the gradient of the Young’s modulus is counterbalanced by the effect due to the vertical stress. Thus, for small |Δ| values, a greater global µ_s_ is observed for Δ < 0, while for larger |Δ| values, this trend is inverted due to the mechanism described previously.

It is remarkable that for Δ < 0, the FEM and SBM simulations predict a very similar behaviour, suggesting that, in this case, two opposite effects are almost cancelled, so that the 2D SBM results provide a good approximation of real values. Although in the previous case, the interplay of effects produced a non-trivial behaviour by varying the gradient, in this case, for Δ < 0, the agreement between FEM and SBM simulations suggests that the reduction of the global static friction with the gradient is approximately linear.

The interplay of effects between the grading and the vertical stress distribution, which are both asymmetric with respect to the sliding direction, causes a non-trivial behaviour of the static friction as a function Δ. The effect of the vertical stress distribution can be reduced by designing a triangular grading, according to Equation 5, so that for Δ > 0 the detachment begins at the centre of the surface and propagates towards the edges, and vice versa for Δ < 0.

**Figure 10:**
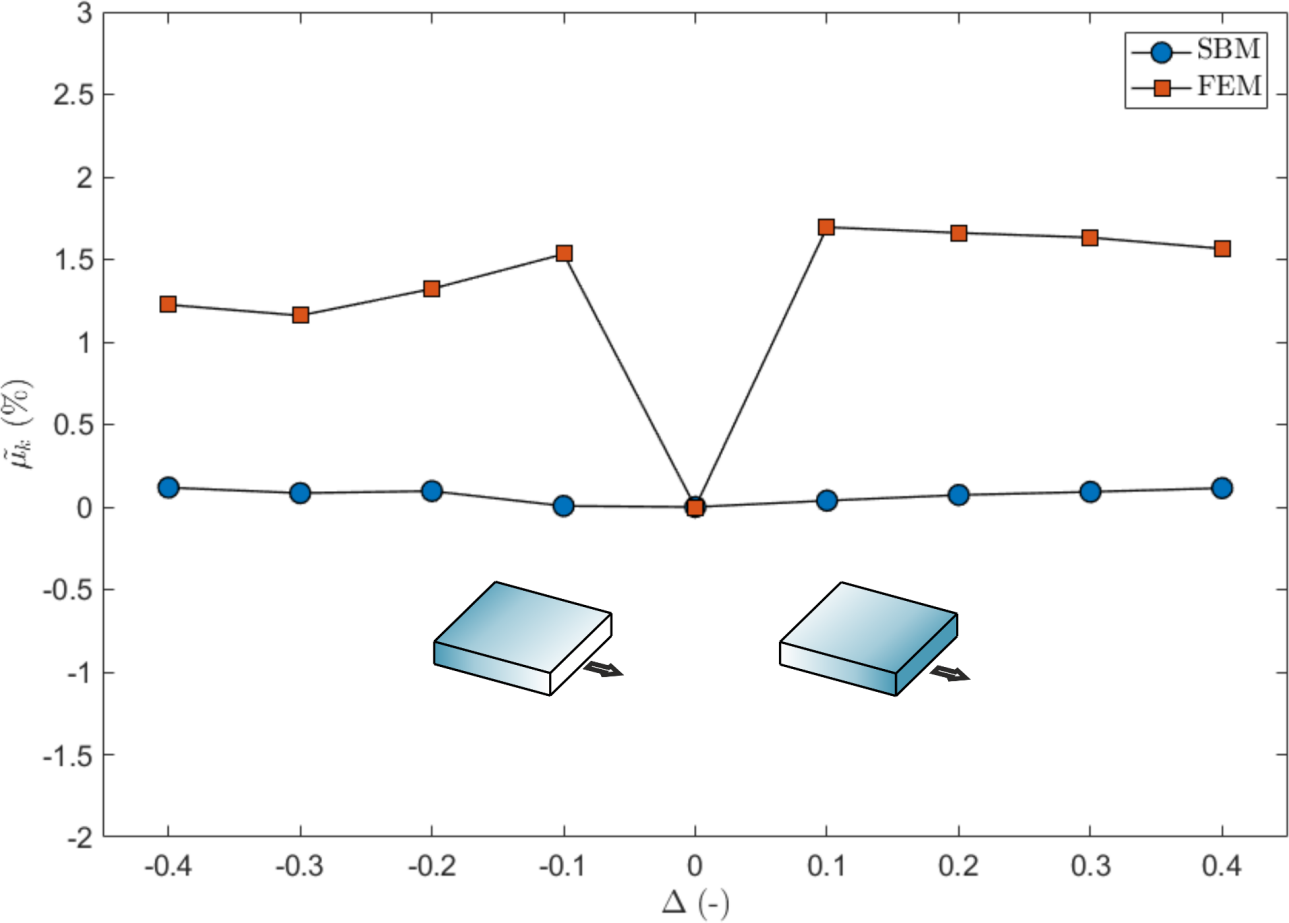
Effect of a gradient in the Young’s modulus on the global dynamic coefficient of friction, expressed as percentage variation as a function of Δ. The shaded blocks represent schematically the value of the Young’s modulus, which is greater for a darker shading.

As in the previous case, no appreciable variation in the dynamic coefficient of friction is predicted, as shown in Figure 10. One difference is that both the SBM and the FEM simulations predict a higher global µ_k_ with respect to the case of non-graded materials. Again, the FEM results are slightly anisotropic as a function of Δ and, as in Figure 7, a smaller global dynamic coefficient of friction is obtained for Δ < 0.

**Figure 11:**
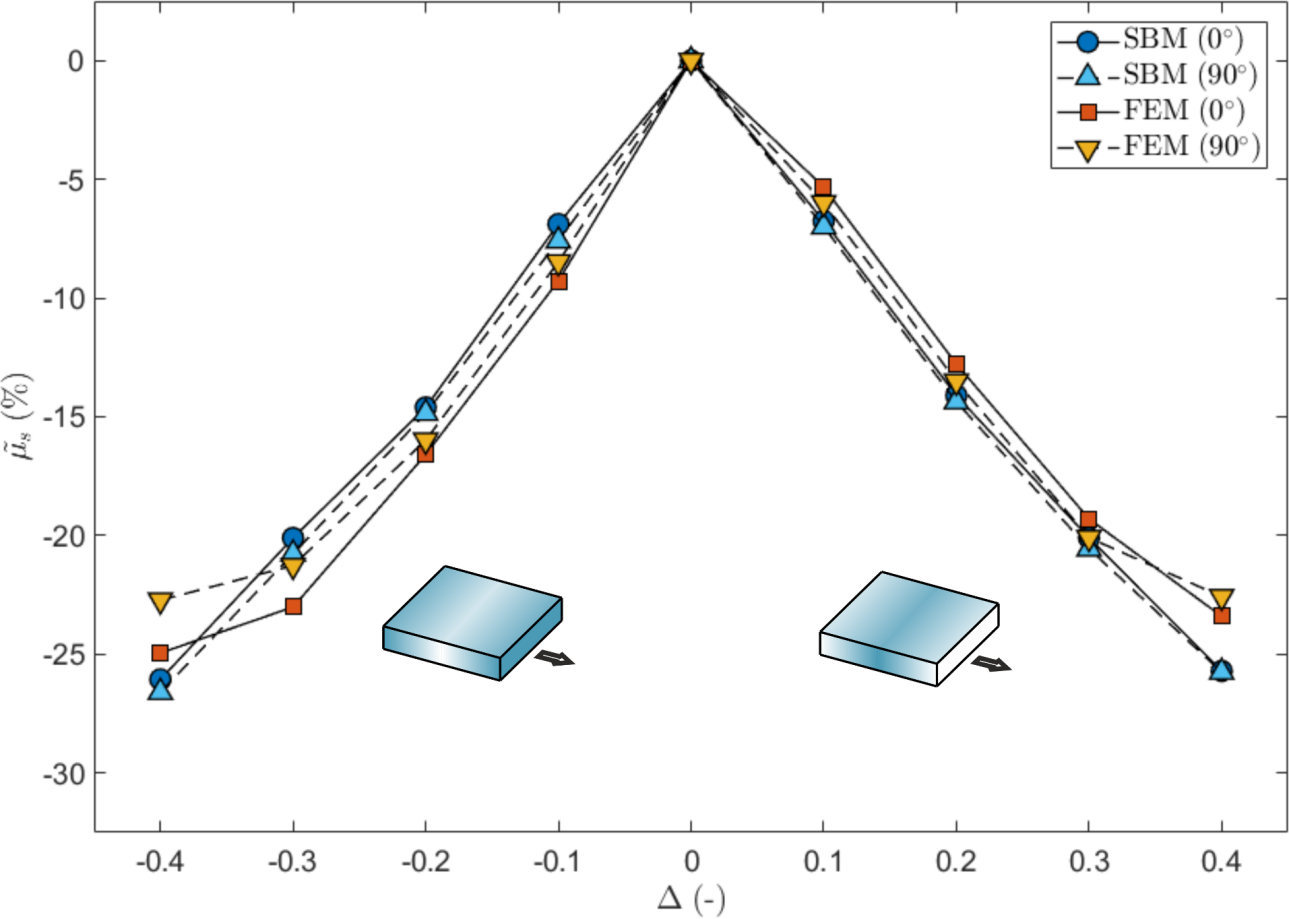
Effect of a triangular gradient in the Young’s modulus on the global static coefficient of friction, expressed as variation as function of Δ and for two sliding directions (0°, i.e., parallel, and 90°, i.e., perpendicular to the gradient). The shaded blocks represent schematically the value of the local Young’s modulus, which is higher for darker shading.

**Figure 12:**
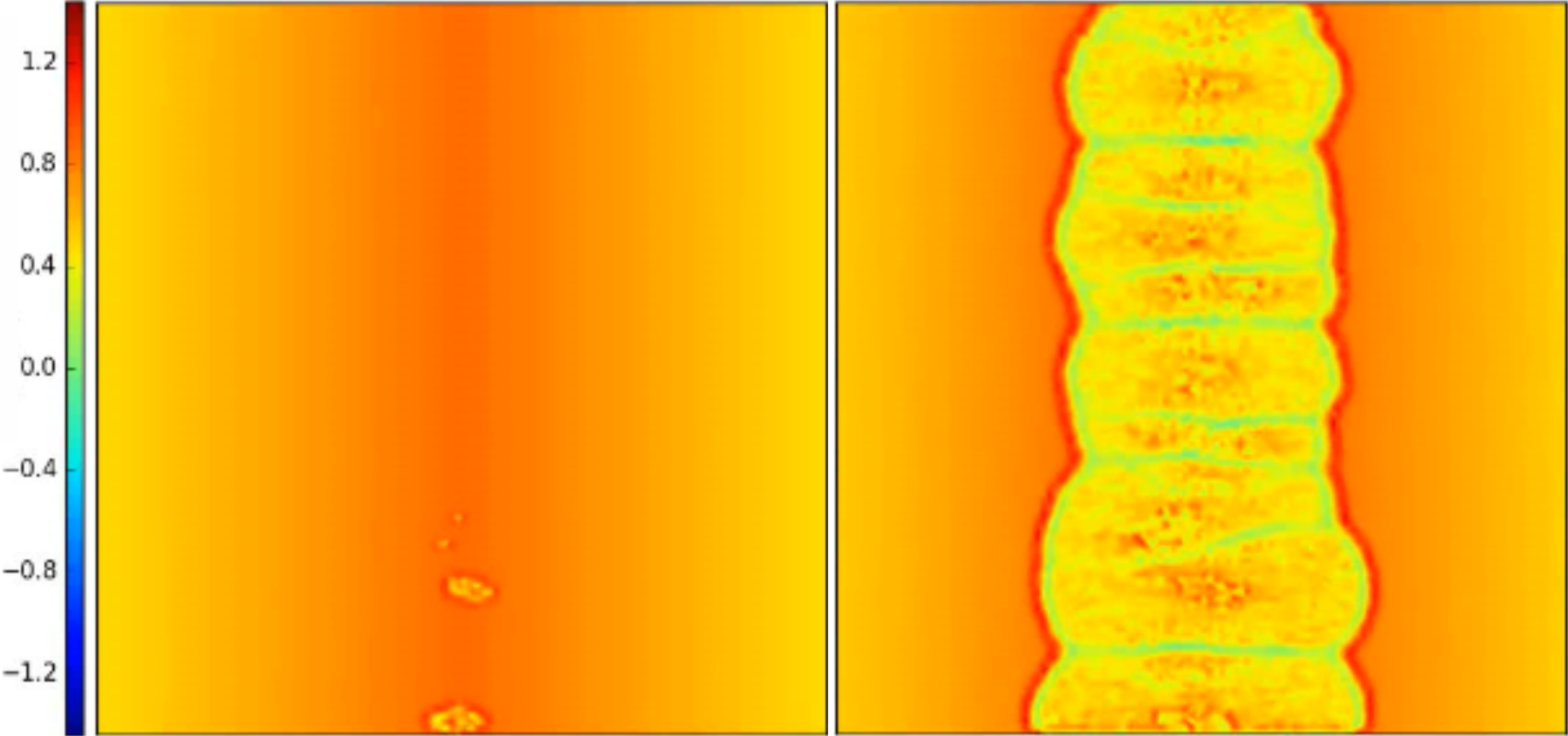
Propagation of the detachment front at the static friction threshold (left) and immediately after (right), in units of the dimensionless longitudinal stress σ*_x_*/*p*, for a material with a triangular gradient in the Young’s modulus, computed using the SBM method for the case Δ = 0.2. The irregularities of the detachment front are due to the statistical dispersion of the local coefficients of friction introduced in the SBM formulation.

The results presented in Figure 11 show the effect of a triangular gradient in the Young’s modulus on the global static coefficient of friction calculated via SBM and FEM. The SBM results display a symmetric behaviour. The corresponding detachment process is shown in Supporting Information File 5 and Supporting Information File 6 for the case Δ > 0 and Δ < 0, respectively (see also Figure 12 for an example). The FEM simulations predict a smaller µ_s_ in the case of Δ < 0 because the effect due to the grading is superimposed on the effect of the vertical stress, so that the static friction thresholds are exceeded earlier compared to the case Δ > 0. However, in both cases, the static friction decreases linearly with Δ. When considering the 90° sliding direction, i.e., orthogonal to the grading, the results of the SBM and the FEM simulations are in good agreement. In this case, the effects due to the vertical stress are ininfluential, since the detachment process is symmetric with respect to the sliding direction. This suggests that with a proper combination of grading and sliding direction, it is possible to obtain a linear reduction of the static friction with the grading level, which would allow the global static friction of a surface to be conveniently tuned to a chosen value, reduced with respect to the corresponding non-graded surface.

## Conclusion

In this paper, we have considered the frictional sliding over a rigid substrate of an elastic material characterized by a grading of selected mechanical properties (Young’s modulus and local coefficients of friction), focusing on the effects on the global static friction and the detachment process at the onset of sliding. The system has been investigated by means of numerical simulations using 2D SBM and 3D FEM to verify the results and to exploit additional insights provided by the two different approaches, after having tuned the SBM parameters in order to have a precise match of the frictional force curve obtained by FEM, in terms of slope and static friction threshold.

The results show that grading of the mechanical properties can reduce the global static friction with respect to a non-graded material, due to an anticipated detachment process. In the case of a graded distribution of local friction coefficients, detachment begins from the region where thresholds on static friction are smaller, while in the case of a graded Young’s modulus distribution, detachment begins from the regions where it is larger. In both cases, the 2D SBM predicts a linear decrease of the global coefficient of friction as a function of the relative grading variation and symmetry with respect to the sliding direction. In contrast, FEM simulations display an anisotropic behaviour due to the effect of the vertical stress distribution, which can either enhance or counterbalance the effect of the grading. Thus, a greater reduction of static friction can be expected when the grading on local friction decreases along the sliding direction, or when a grading of Young’s modulus increases along the sliding direction. The effect on the global dynamic coefficient of friction, instead, appears to be underestimated numerically, and should be the object of further investigations.

These results are not valid when the relative grading variation is greater than 30% with respect to the average. In this case, the time evolution of the tangential force changes radically. The time duration of the detachment phase increases due to the large variation between edges so that the force peak in the transition from static to dynamic friction can disappear completely.

This interplay of effects produces a nonlinear reduction of static friction with the grading. A quasi-linear decrease can be obtained in the case of a triangular grading, which is symmetric with respect to the two edges, so that the anisotropy of the vertical stress distribution is less influential. In particular, this outcome can be achieved by setting this type of grading along the orthogonal direction with respect to the sliding direction.

We have thus found that the SBM can capture the main effects of gradings on the static friction coefficient and describe the detachment process at the interface, with a much smaller computational cost than that required by FEM simulations. Therefore, it can be adopted for a rapid, initial estimation of static friction values.

These results suggest that it is possible to realize bio-inspired materials with a gradient in the mechanical properties, imitating the graded Young’s moduli found in nature, or in the local frictional properties, e.g., by controlling the roughness or the microstructure, for the design of advanced sliding interfaces. A reduction in the static friction up to almost 30%, with respect to the corresponding non-graded material, can thus be achieved.

## Supporting Information

File 1 Detachment of a surface with a small gradient in the local coefficients of friction.
Time evolution of the dimensionless longitudinal stress σ*_x_*/*p* for a surface with a gradient in the local coefficients of friction, computed using SBM for the case Δ = 0.1. The detachment process starts where the local static friction threshold is smaller, i.e., where the local static coefficient of friction is smaller.

File 2 Detachment of a surface with a large gradient in the local coefficients of friction.
Time evolution of the dimensionless longitudinal stress σ*_x_*/*p* for a material with a gradient in the local coefficients of friction, computed using SBM for the case Δ = 0.3. The detachment process starts where the local static friction threshold is smaller, i.e., where the local static coefficient of friction is smaller.

File 3 Detachment of a surface with a positive triangular gradient in the material Young’s modulus.
Time evolution of the dimensionless longitudinal stress σ*_x_*/*p* for a material with a triangular gradient in the Young’s modulus, computed using SBM for the case Δ = 0.2. The detachment process starts at the centre of the sliding plate and propagates towards the edges.

File 4 Detachment of a surface with a negative triangular gradient in the material Young’s modulus.
Time evolution of the dimensionless longitudinal stress σ*_x_*/*p* for a material with a negative triangular gradient in the Young’s modulus, computed using SBM for the case Δ = −0.2. The detachment process starts at the edges of the sliding plate and propagates towards the centre.

File 5 Effect of the finite-element type on the surface stress distributions.
Normalised normal and tangential stresses with respect to the applied pressure *p* as a function of the dimensionless coordinate *x*/*L*. C3D8R: 8-node brick with reduced integration, C3D8I: 8-node brick with 8 points of integration and incompatible modes.

File 6 Computed values of the global static coefficients of friction.
Tables reporting the computed absolute values of the global static coefficients of friction µ_s_, as function of Δ, obtained using SBM and FEM, for the case of a gradient in the local coefficients of friction and a gradient in the material Young’s modulus.
