# Supplementary material for: Evidence of friction reduction in laterally graded materials
Source: Beilstein J Nanotechnol. 2018 Sep 13;9:2443–56. doi: 10.3762/bjnano.9.229 (PMC6142729; doi:10.3762/bjnano.9.229)
Supplement: File 5 — Effect of the finite-element type on the surface stress distributions. Normalised normal and tangential stresses with respect to the applied pressure p as a function of the dimensionless coordinate x/L. C3D8R: 8-node brick with reduced integration, C3D8I: 8-node brick with 8 points of integration and incompatible modes. [file Beilstein_J_Nanotechnol-09-2443-s005.pdf]

Supporting Information

for

Evidence of friction reduction in laterally graded materials

Roberto Guarino[1], Gianluca Costagliola[2], Federico Bosia[2], and Nicola Maria Pugno[1,3,4,*]

Address: [1]Laboratory of Bio-Inspired & Graphene Nanomechanics, Department of Civil, Environmental and Mechanical Engineering, University of Trento, Via Mesiano 77, 38123 Trento, Italy, [2]Department of Physics and Nanostructured Interfaces and Surfaces Centre, University of Torino, Via Pietro Giuria 1, 10125 Torino, Italy, [3]Ket Lab, Edoardo Amaldi Foundation, Italian Space Agency, Via del Politecnico snc, 00133 Rome, Italy and [4]School of Engineering and Materials Science, Queen Mary University of London, Mile End Road, E1-4NS London, United Kingdom

[*] Corresponding author

Email: Nicola Maria Pugno - nicola.pugno@unitn.it

Effect of the finite-element type on the surface stress distributions

Normalised normal and tangential stresses with respect to the applied pressure *p* as function of the dimensionless coordinate *x*/*L*. C3D8R: 8-node brick with reduced integration, C3D8I: 8-node brick with 8 points of integration and incompatible modes.

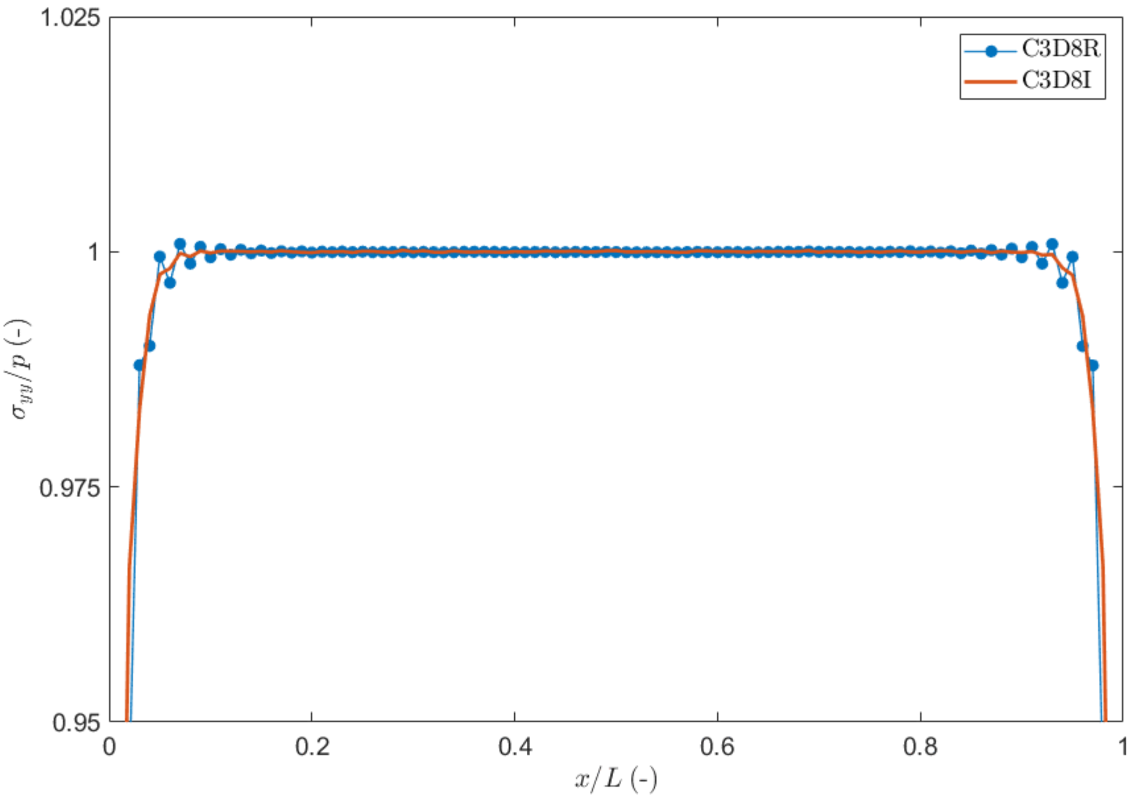

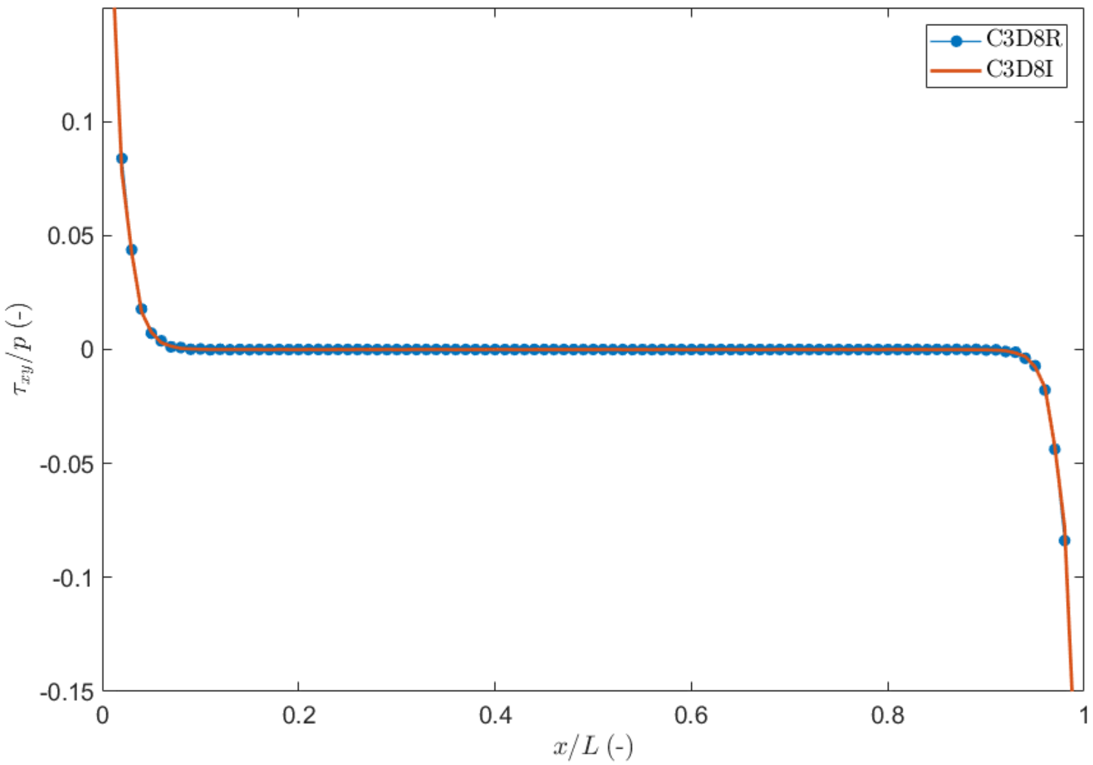

C3D8R
C3D8I
0.1
0.05
0
-0.05
-0.1
-0.15
0
0.2
0.4
0.6
0.8
1
τ_{xy}/p (-)
x/L (-)
