# Supplementary material for: Evidence of friction reduction in laterally graded materials
Source: Beilstein J Nanotechnol. 2018 Sep 13;9:2443–56. doi: 10.3762/bjnano.9.229 (PMC6142729; doi:10.3762/bjnano.9.229)
Supplement: File 6 — Computed values of the global static coefficients of friction. Tables reporting the computed absolute values of the global static coefficients of friction µs, as function of Δ, obtained using SBM and FEM, for the case of a gradient in the local coefficients of friction and a gradient in the material Young’s modulus. [file Beilstein_J_Nanotechnol-09-2443-s006.pdf]

# Supporting Information
# for

# Evidence of friction reduction in laterally graded materials

Roberto Guarino[1], Gianluca Costagliola[2], Federico Bosia[2], and Nicola Maria Pugno[1,3,4,*]

Address: [1]Laboratory of Bio-Inspired & Graphene Nanomechanics, Department of Civil, Environmental and Mechanical Engineering, University of Trento, Via Mesiano 77, 38123 Trento, Italy, [2]Department of Physics and Nanostructured Interfaces and Surfaces Centre, University of Torino, Via Pietro Giuria 1, 10125 Torino, Italy, [3]Ket Lab, Edoardo Amaldi Foundation, Italian Space Agency, Via del Politecnico snc, 00133 Rome, Italy and [4]School of Engineering and Materials Science, Queen Mary University of London, Mile End Road, E1-4NS London, United Kingdom

[*] Corresponding author

Email: Nicola Maria Pugno - nicola.pugno@unitn.it

# Computed values of the global static coefficients of friction

Tables reporting the computed absolute values of the global static friction coefficient $\mu_s$, as function of Δ and obtained through SBM and FEM, for the case of gradients in the local friction coefficients and gradients in the material Young's modulus.

| **Gradients in the local coefficients of friction** | | |
|---|---|---|
| Δ | SBM | FEM |
| −0.4 | 0.6198 | 0.7080 |
| −0.3 | 0.6501 | 0.7128 |
| −0.2 | 0.7458 | 0.7321 |
| −0.1 | 0.8346 | 0.8237 |
| 0.0<br>(reference case) | 0.9124 | 0.9124 |
| 0.1 | 0.8336 | 0.8640 |
| 0.2 | 0.7442 | 0.7776 |
| 0.3 | 0.6491 | 0.6835 |
| 0.4 | 0.6212 | 0.6746 |

| Gradients in the material Young's modulus | | |
|---|---|---|
| Δ | SBM | FEM |
| −0.4 | 0.6725 | 0.6844 |
| −0.3 | 0.7236 | 0.7197 |
| −0.2 | 0.7789 | 0.7756 |
| −0.1 | 0.8509 | 0.8451 |
| 0.0 (reference case) | 0.9124 | 0.9124 |
| 0.1 | 0.8494 | 0.8325 |
| 0.2 | 0.7808 | 0.7667 |
| 0.3 | 0.7251 | 0.7500 |
| 0.4 | 0.6754 | 0.7278 |
